# Supplementary material for: The Regulatory Mechanism of Transthyretin Irreversible Aggregation through Liquid-to-Solid Phase Transition
Source: Int J Mol Sci. 2023 Feb 13;24(4):3729. doi: 10.3390/ijms24043729 (PMC9960511; doi:10.3390/ijms24043729)
Supplement: Supplementary file 1 [file ijms-24-03729-s001.zip › ijms-2163869-supplementary.pdf]

Supporting Information

The regulatory mechanism of transthyretin irreversible aggregation through liquid-to-solid phase transition

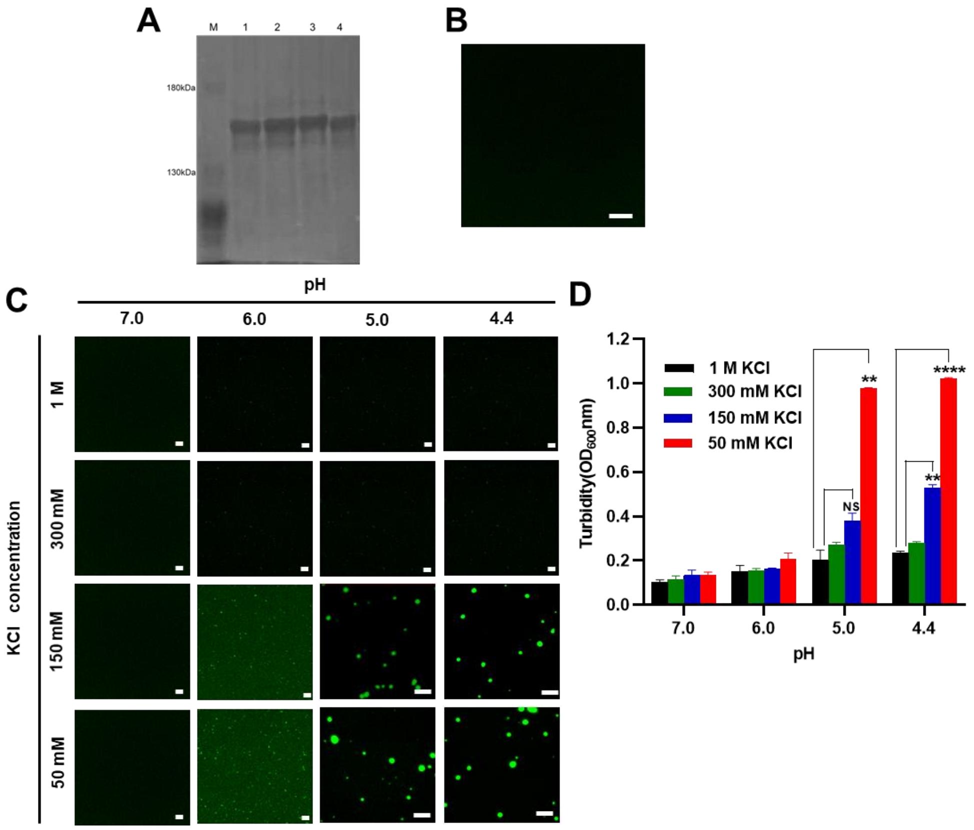

Figure S1. Influencing EGFP-TTR phase separation

(**A**) The native-PAGE of EGFP-TTR and mutant proteins. M: marker; 1: EGFP-TTR; 2: EGFP-TTR(V30M); 3: EGFP-TTR(R34T); 4: EGFP-TTR(K35T). (**B**) Confocal microscope of 10 μM EGFP at pH 4.4. Scale bar, 5 μm. (**C**) Confocal microscope images of 10 μM EGFP-TTR at varying pH and salt concentrations. Scale bar, 2.5 μm. (**D**) Turbidity of 10 μM EGFP-TTR at varying pH and salt concentrations. Error bars represented SEM (n=3). ****P≤0.0001, ***P≤0.001, **P≤0.01, *P≤0.05. The P values for 150 mM KCl and 50 mM KCl were 0.0882 and 0.0061, respectively, with respect to 10 μM EGFP-TTR (pH 5.0, 1 M KCl). The P values for 150 mM KCl and 50 mM KCl were 0.0027 and 0.0001, respectively, with respect to 10 μM EGFP-TTR (pH 4.4,1 M KCl).

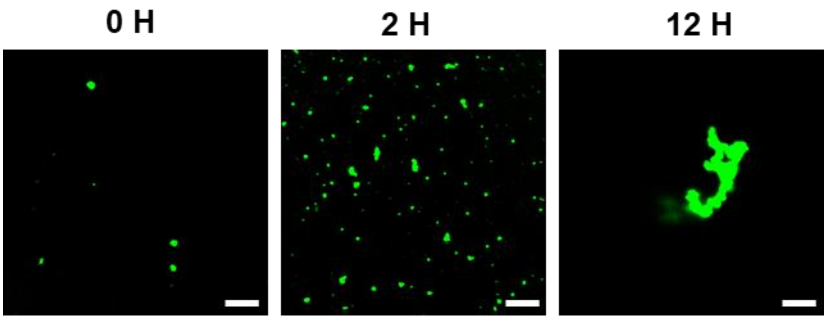

Figure S2. the effect of EGFP tag on TTR transition

Time-dependent changes of EGFP-TTR liquid droplets analyzed. 10 μM EGFP-TTR were incubated at pH 4.4, 37 °C. Scale bar, 5 μm.

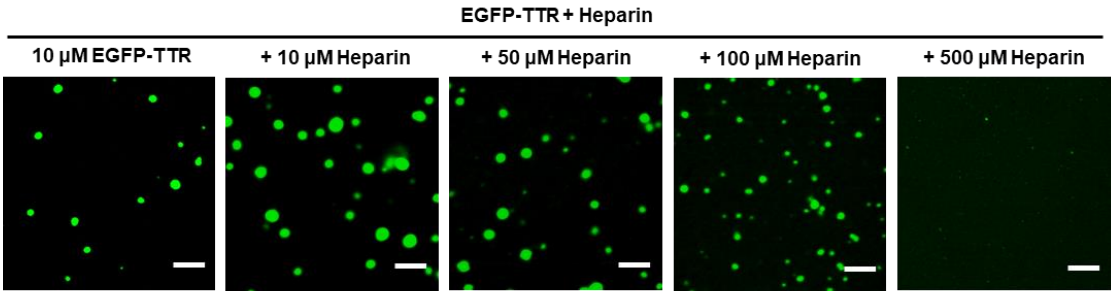

Figure S3. The effect of heparin on EGFP-TTR phase separation

Confocal microscope images of 10 μM EGFP-TTR in the presence of varying heparin concentrations at pH 4.4. Scale bar, 2.5 μm.

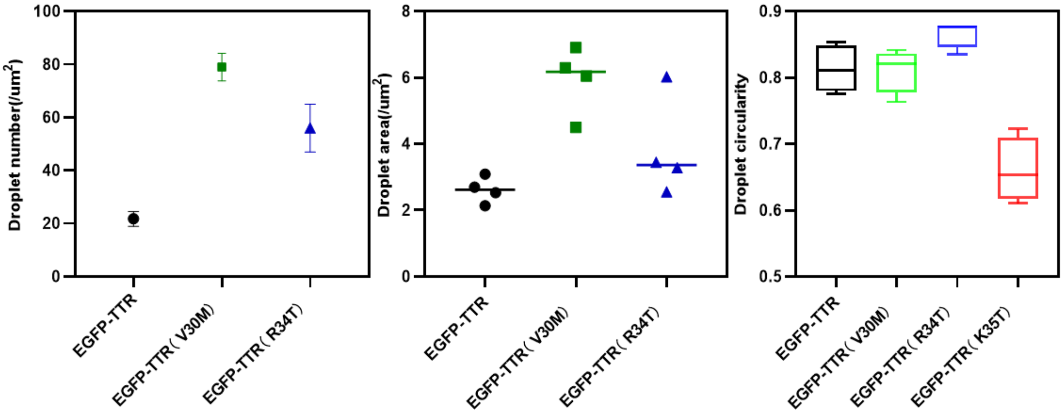

Figure S4.

Droplet number (left), area (middle) and circularity (right) analyses of figure 3A were shown. Error bars represented SEMs. The results were representative of three biological replicates.

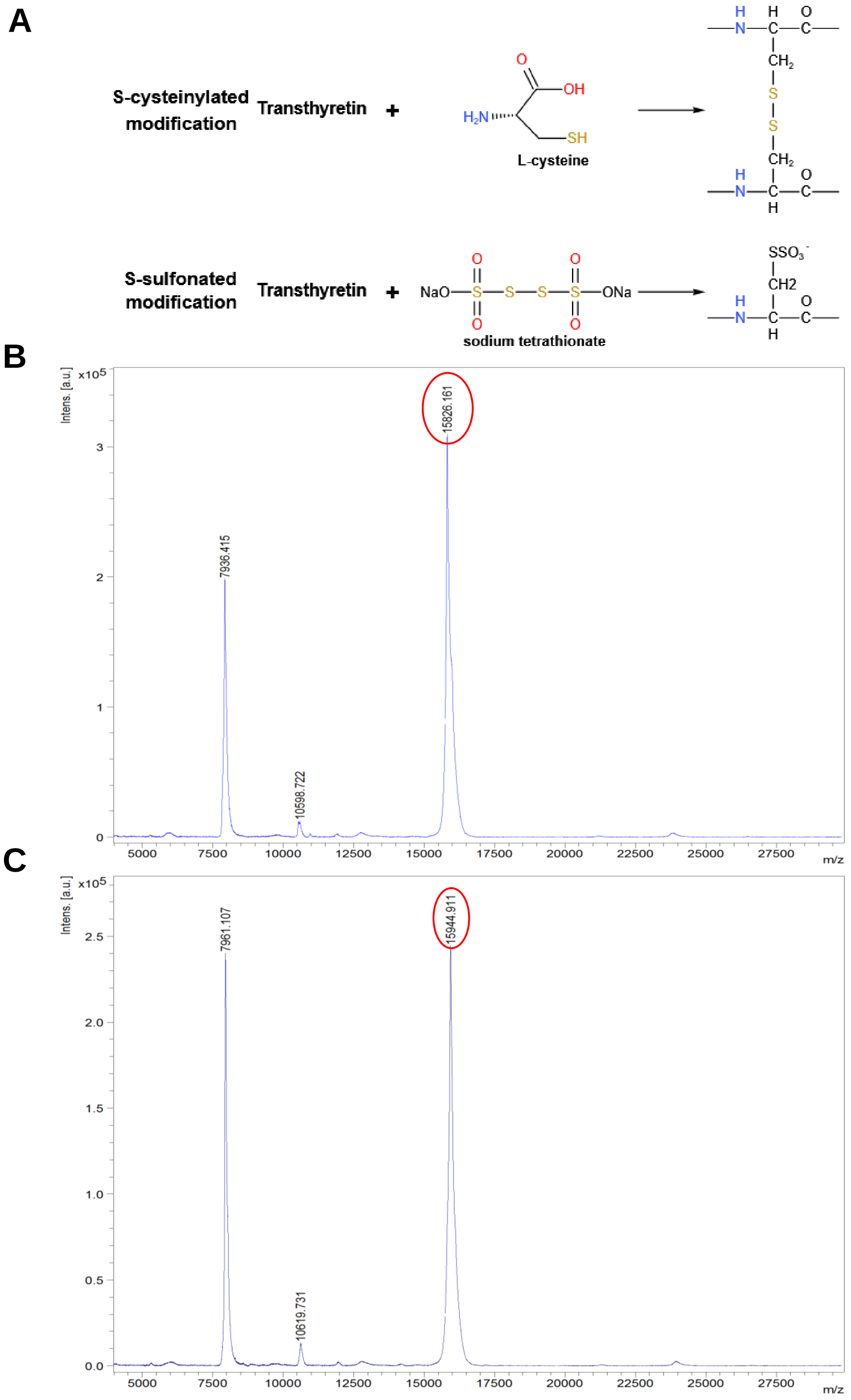
A
S-cysteinylated modification
Transthyretin
+
L-cysteine
S-sulfonated modification
Transthyretin
+
sodium tetrathionate
B
Intens. [a.u.]
x10^5
7936.415
10598.722
15826.161
m/z
C
Intens. [a.u.]
x10^5
7961.107
10619.731
15944.911
m/z

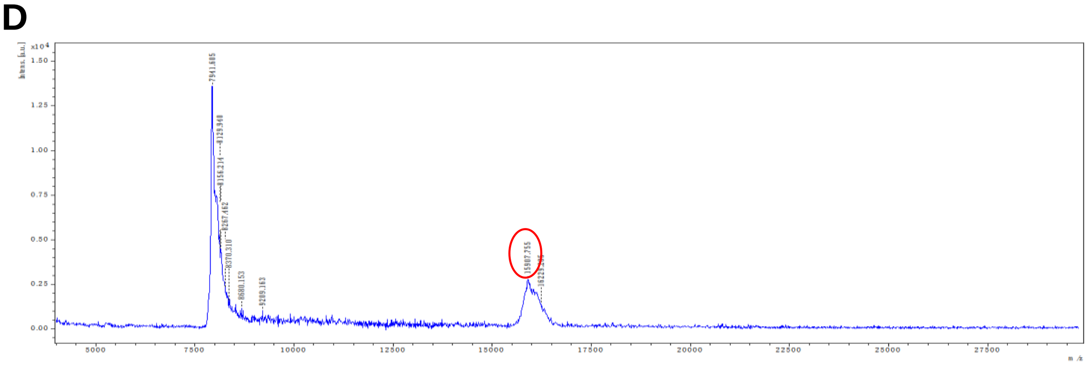

Figure S5. Oxidative modification method and identification of TTR

(**A**) Oxidative method for S-cysteinylation and S-sulfonation of TTR. (**B**) MALDI-TOF MS spectra of untreated TTR. Mr: 15826.161 Da. (**C**) MALDI-TOF MS spectra of S-cysteinylated TTR. Mr:15944.911 Da. (**D**) MALDI-TOF MS spectra of S-sulfonated TTR. Mr:15906 Da

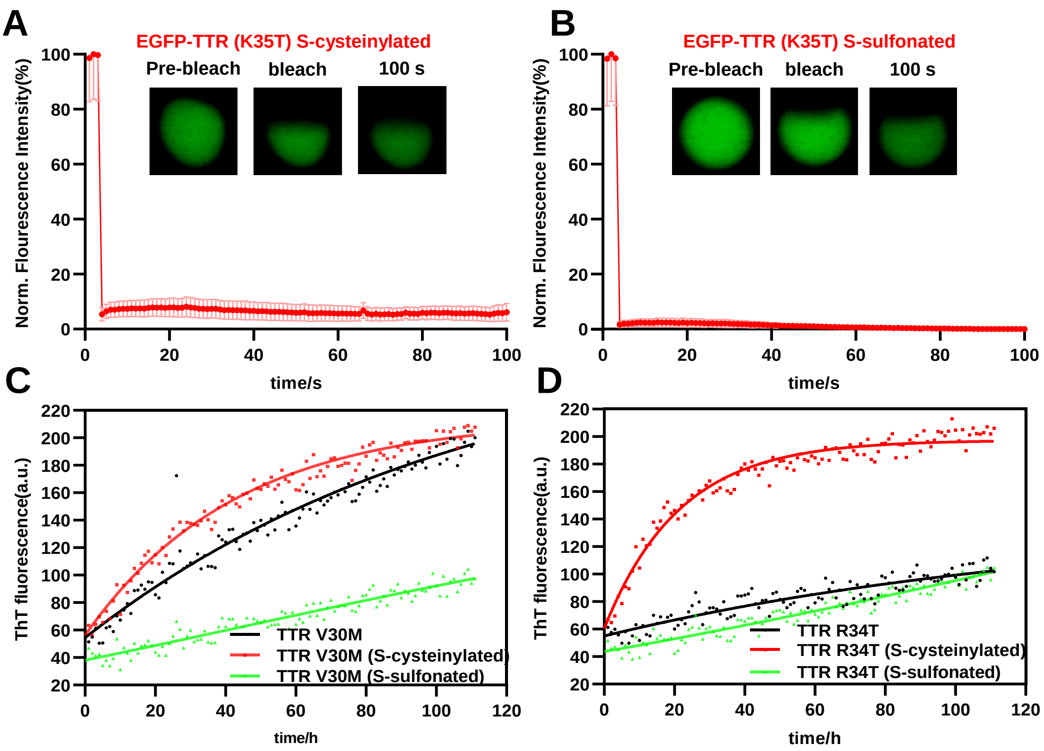

Figure S6. S-cysteinylated and S-sulfonated of TTR

(**A**) FRAP of 10 μM S-cysteinylated EGFP-TTR K35T at pH 4.4. Error bars represented SEM (n=3). (**B**) FRAP of 10 μM S-sulfonated EGFP-TTR K35T at pH 4.4. Error bars represented SEM (n=3). (**C**) The ThT fluorescence intensity traced for 40 μM S-cysteinylated and S-sulfonated TTR V30M at pH 4.4, 37°C. (**D**) The ThT fluorescence intensity traced for 40 μM S-cysteinylated and S-sulfonated TTR R34T at pH 4.4, 37°C.
